# *DMD* Mutations in 576 Dystrophinopathy Families: A Step Forward in Genotype-Phenotype Correlations

**DOI:** 10.1371/journal.pone.0135189

**Published:** 2015-08-18

**Authors:** Jonas Juan-Mateu, Lidia Gonzalez-Quereda, Maria Jose Rodriguez, Manel Baena, Edgard Verdura, Andres Nascimento, Carlos Ortez, Montserrat Baiget, Pia Gallano

**Affiliations:** 1 Genetics Department, Hospital de la Santa Creu i Sant Pau, U705 CIBERER, Barcelona, Spain; 2 Genetics Department, Hospital de la Santa Creu i Sant Pau, Barcelona, Spain; 3 Neuromuscular Unit, Hospital Sant Joan de Deu, Esplugues de Llobregat, Spain; University of Minnesota, UNITED STATES

## Abstract

Recent advances in molecular therapies for Duchenne muscular dystrophy (DMD) require precise genetic diagnosis because most therapeutic strategies are mutation-specific. To understand more about the genotype-phenotype correlations of the *DMD* gene we performed a comprehensive analysis of the *DMD* mutational spectrum in a large series of families. Here we provide the clinical, pathological and genetic features of 576 dystrophinopathy patients. *DMD* gene analysis was performed using the MLPA technique and whole gene sequencing in blood DNA and muscle cDNA. The impact of the DNA variants on mRNA splicing and protein functionality was evaluated by *in silico* analysis using computational algorithms. DMD mutations were detected in 576 unrelated dystrophinopathy families by combining the analysis of exonic copies and the analysis of small mutations. We found that 471 of these mutations were large intragenic rearrangements. Of these, 406 (70.5%) were exonic deletions, 64 (11.1%) were exonic duplications, and one was a deletion/duplication complex rearrangement (0.2%). Small mutations were identified in 105 cases (18.2%), most being nonsense/frameshift types (75.2%). Mutations in splice sites, however, were relatively frequent (20%). In total, 276 mutations were identified, 85 of which have not been previously described. The diagnostic algorithm used proved to be accurate for the molecular diagnosis of dystrophinopathies. The reading frame rule was fulfilled in 90.4% of DMD patients and in 82.4% of Becker muscular dystrophy patients (BMD), with significant differences between the mutation types. We found that 58% of DMD patients would be included in single exon-exon skipping trials, 63% from strategies directed against multiexon-skipping exons 45 to 55, and 14% from PTC therapy. A detailed analysis of missense mutations provided valuable information about their impact on the protein structure.

## Background

Dystrophinopathies are the most common forms of muscular dystrophy in childhood. They are caused by mutations in the X chromosome-linked *DMD* gene [OMIM: *300377] [1, 2]. *DMD* gene is the largest known human gene, spanning 2.22 Mb in the region Xp21, and it has 79 exons and 8 promoters. In addition, most of these small mutations are unique and one-third are sporadic. This gene encodes the protein called dystrophin, a key element in stabilizing the sarcolemma during muscle contraction [3]. There are two main phenotypes: Duchenne muscular dystrophy, a severe form that has an incidence of 1:3500 male births [OMIM # 310200], and Becker muscular dystrophy, a mild form with an incidence of 1:20000 male births, [OMIM # 300376]. One-third of the mutations are *de novo* [4]. Clinical severity depends on whether or not the reading frame of the gene is maintained: DMD is mostly caused by out-frame mutations while BMD is caused by in-frame mutations [5]. Mutations in the *DMD* gene can be associated with X-linked dilated cardiomiopathy, in which case patients present only with heart problems. [OMIM # 302045], [6].

Since the gene was discovered in 1986, many mutations have been described. Mutational studies have traditionally focused on the detection of exonic deletions, representing 65% of all mutations. The observation that some exons were more frequently deleted led to the implementation of multiplex PCR as a standard diagnostic method [7]. This technique can detect up to 98% of deletions, but it does not delimit the extent of the deletions or detect exonic duplications, which represent up to 10% of the mutations. Moreover, multiplex PCR is unable to detect mutations in carrier women. To detect duplications, it is necessary to analyse the number of exonic copies. The recent introduction of dosimetry methods based on PCR MLPA (multiplex ligation-dependent probe amplifcication) has significantly improved the detection of large intragenic rearrangements in the 79 exons that constitute the gene [8, 9].

The remaining mutations are small mutations and they account for approximately 25–30% of the molecular pathology of the *DMD* gene. The analysis of these small mutations has historically been a difficult task, mainly due to the gene’s size. The search for rapid and economical detection of these mutations led to the development of several techniques [10–13]. In recent years, the affordable cost of Sanger sequencing and the development of massive sequencing systems (Next Generation Seguencing, NGS) has made *DMD* gene analysis affordable. Flanigan and co-workers described a semi-automatic technique for direct sequencing of the 79 exons and flanking intron sequences in genomic DNA called SCAIP (single-condition amplification/internal first) [14]. This technique, that also detects deletions, has a higher sensitivity than the screening methods based on conformational analysis or heteroduplex. In 2007, Deburgrave et al. described another diagnostic strategy based on the use of muscle biopsy, combining protein analysis by Western-blotting and multiplex mutational study by direct sequencing of mRNA [15]. This technique is highly efficient, since the detection rate is almost 100% in patients with altered quantitative or qualitative Western-blot for dystrophin. Furthermore, it can detect virtually all types of mutations. It provides a comprehensive correlation between genotype and protein expression, helping to predict clinical phenotype. In this paper we describe 576 dystrophinopathy families and discuss the mutational spectrum associated with the *DMD* gene and its impact on the dystrophin structure.

## Materials and Methods

### Patients

Patients with DMD/BMD were grouped into four categories: Duchenne muscular dystrophy (DMD), Becker muscular dystrophy (BMD), intermediate muscular dystrophy (IMD), and pure cardiac X-linked dilated cardiomyopathy (XLCM), based on clinical presentation, family history, age of onset of symptoms, disease progression, and age of loss of ambulation (DMD <13, BMD ≥16, IMD ≥13 and XLCM <16). To avoid bias, we included only one case for each family. A histopathological study was performed prior to molecular analysis in all cases for which a muscle biopsy was available. Dystrophin expression was analysed using monoclonal antibodies against N-terminal epitopes (DYS3), rod domain (DYS1) and C-terminus (DYS2) (Novocastra, Newcastle upon Tyne, UK). Other sarcolemmal proteins, such as sarcoglycans α, β, γ and δ, caveolin-3, dysferlin, utrophin and emerin, were also analysed by IHC.

Written informed consent was obtained from all patients. In those cases where patients were minors, written informed consent was authorised and signed by their parents. Adult patients authorised and signed their own informed consent. The studies were approved by the Ethics Committee at Hospital de la Santa Creu I Sant Pau (HSPSC).

### Genetic analysis

DNA from patients and their relatives was obtained from samples extracted from peripheral blood according to standard procedures. Before studying the small mutations, we analysed exonic deletions and duplications using MLPA of the 79 *DMD* exons (P034 and P035 Sauce Kit, MRC-Holland) following the manufacturer's instructions. If a deletion of a single exon was identified, an alternative method was required (PCR, QF-PCR or real-time qPCR, exon sequencing) to rule out a point mutation, rare variant or polymorphism altering the normal MLPA pattern. Small mutations were detected in most patients by direct sequencing of the 79 exons and flanking intron regions by SCAIP [14]. When muscle tissue was available, mutational analysis was performed on cDNA and subsequently confirmed in genomic DNA. Muscle mRNA was extracted using 30 mg of muscle with the RNeasy Fibrous Tissue Mini Kit (Qiagen, Hilden, Germany) and retrotranscribed to cDNA by RT-PCR using polythiamine primers (Invitrogen, Carlsbad, USA). The full *DMD* transcript was amplified and sequenced in 20 overlapping amplicons with our own primers and others described previously [15]. Sequence analysis was performed by Sanger sequencing method (Big Dye 3.1 chemistry and equipment 3500xL ABI, Applied Biosymtems, Foster City, USA).

The impact of the DNA variants detected on mRNA splicing and protein functionality was evaluated by *in silico* analysis using computational algorithms. Altered splicing sites were analyzed using positional weight matrices included in the Human Splicing Finder and Alamut softwares. Pathogenicity of amino acid substitutions was evaluated using Polyphen-2 [16] and SIFT [17] algorithms. All substitutions with a Polyphen-2 score below 1 and a SIFT score below 0.05 were considered damaging substitutions. Moreover, when an aminoacid substitution was detected in any of the 100 healthy controls, pathogenicity was rejected. In addition, we used LOVD database (Leiden Open Variation Database: www.dmd.nl) as a reference to determine if the substitution consisted in a missense mutation or a non-synonymous SNP. The nucleotide position was determined according to the *DMD* reference sequence (RefSeq NM_004006.2) and mutation nomenclature followed the guidelines of the Human Genome Variation Society (HGVS).

The mutation rate per nucleotide and per generation for each mutation type was calculated according to the formula μx = mn_x_ / Nt_x_, as previously described [18].

MARCOIL program was used to predict the existence and location of potential coiled-coil domains in protein sequences[19].

## Results

**Fig 1:**
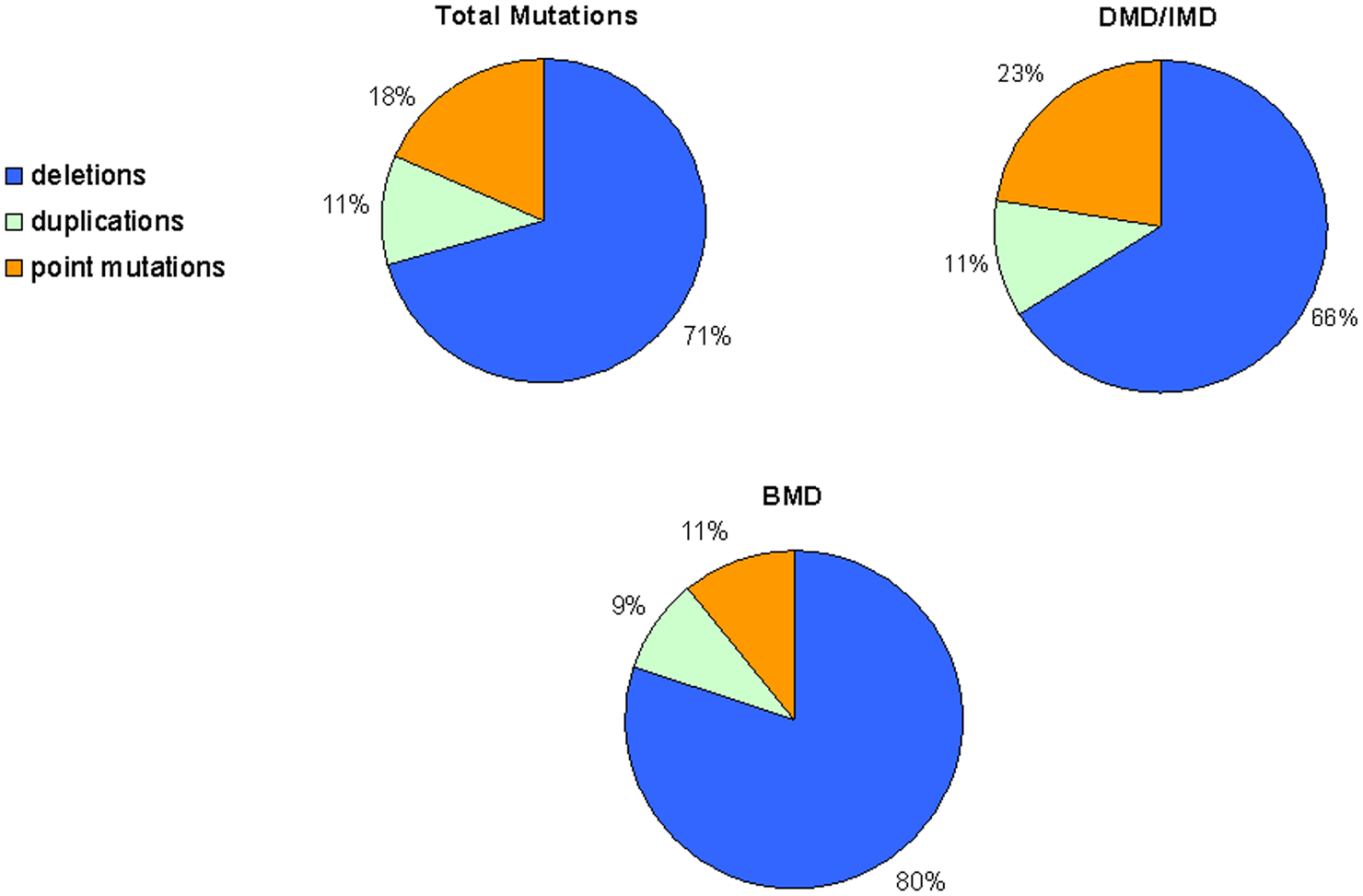
Identified mutations and phenotypic groups.

We identified *DMD* mutations in 576 unrelated families, 564 of which affected males (360 DMD, 7 IMD and 176 BMD) and 12 affected isolated symptomatic female carriers. We were unable to assign 21 male patients to a phenotypic category, either because clinical data were lacking or because the patients were too young. Of the 576 independent mutational events, 471 (81.8%) were major rearrangements (exonic deletions and duplications) and 105 (18.2%) were small mutations. The frequency of deletions, duplications and small mutations differed depending on the phenotypic group. BMD patients had more deletions (80%, 141/176) than DMD/IMD patients (66%, 242/367), while the frequency of mutations in BMD patients was lower (9.1%, 16/176) than that in DMD/IMD patients (22.6%, 83/267) (Fig 1, Table 1).

**Table 1:** Number of identified mutations.

Type of mutation	DMD/IMD	BMD	DMD/BMD	Isolated carrier	Total	%
**Exonic deletion**	242	141	18	5	406	70.5%
In-frame	18	127	15		158	
Frameshift	220	14	3	5	244	
Others	4				4	
**Exonic duplication**	41	19	3	1	64	11.1%
In-frame	7	12	2	1	22	
Frameshift	34	7	1		42	
Others					0	
**Complex rearrangements**	1				1	0.2%
**Nonsense**	46	5		3	54	9.4%
UGA	19	3			22	
UAG	15	1		1	17	
UAA	12	1		2	15	
**Indels**	21	3		2	26	
Frameshift insertion	6	1			7	
Frameshift deletion	11	2		1	14	
Framshift indel	3			1	4	
In-frame deletion	1				1	
**Missense**	3	1			4	0.7%
**Splice Site**	13	6		1	20	3.5%
**Pseudoexon**		1			1	0.2%
**Total Mutations**	367	176	21	12	576	100%

### Exonic deletions

**Fig 2:**
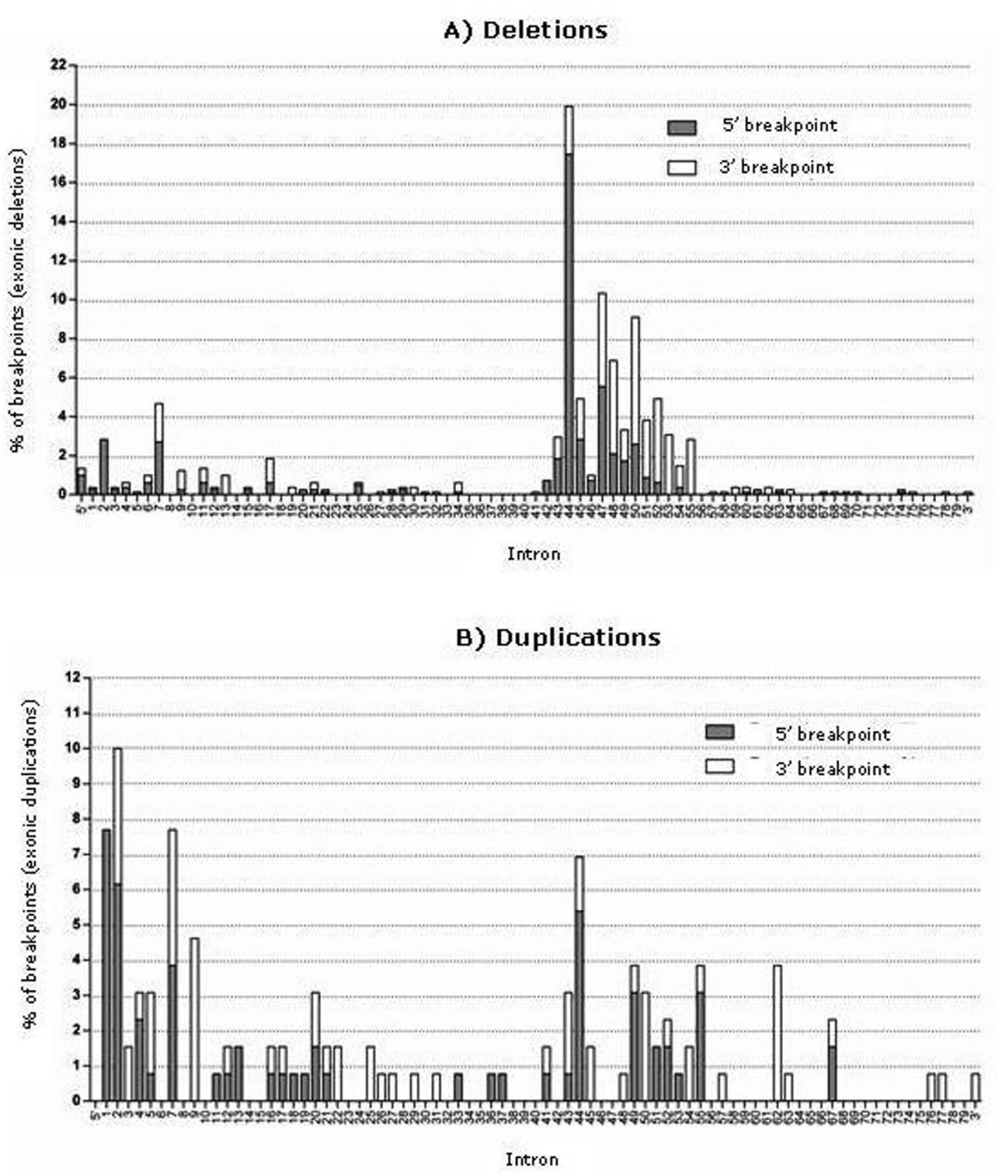
Distribution of intronic breakpoints in large intragenic rearrangements in the *DMD* gene. (A) Breakpoints in deletions. (B) Breakpoints in duplications. 5’ breakpoints in grey and 3’ breakpoints in white.

We identified 471 (81.8%) intragenic rearrangements, 406 (70.5%) of which were deletions and 64 (11.1%) duplications. In one DMD patient, we detected two apparently independent rearrangements (Del Pb>29 and Dup 37>43; S1 Fig). Both deletions and duplications had a non-random distribution with two hot spots. We found that 73.2% (298/407) of deletions started between introns 43 and 55 (distal hot spot), whereas 18.7% (72/407) started between introns 1 and 20 (proximal hot spot). This shows that deletion breakpoints were mainly concentrated in a few introns at the distal hot spot, while proximal breakpoints were more dispersed (Fig 2). Considering the 814 breakpoints, intron 44 was clearly that most frequently involved in deletions (19.9%, 162/814) and mostly as a 5' breaking point (87.6%), followed by introns 47 (10.3%, 84/814) and 50 (9.1%, 74/814). The proportion of 3’ breakpoints respect to 5’ breakpoints tended to gradually increase from intron 46 to intron 55. In the proximal region of the gene, introns 7 (4.7%, 38/814) and 2 (2.8%, 23/814) were the most involved. Intron 2 only presented one 5’ breakpoint while intron 7 equally presented 5’ and 3’ breakpoints. The large length of introns 2, 7 and 44 (170 Kb, 110 Kb and 248 Kb, respectively) must be taken into account when considering the frequency of breakpoints.

Deletions affecting only regions outside the two hot spots were unusual: 4.4% (18/407) were located between the two hot spots (introns 21–42), 2% (8/407) starting at 5' end of *DMD* gene, and 1.7% were located between introns 55 and 79. Only seven cases showed deletions that included the two hot spots (1.7%). Furthermore, 8 deletions started in regions at 5' end of exon 1, deleting brain and muscle promoters. One of them, associated with the DMD phenotype, deleted the entire *DMD* gene and 5’ flanking genes (*GK* [OMIM: *300474] and *OTC* [OMIM: *300461]). Three deletions associated with DMD were limited to the promoter region.

In our cohort of mutations, 131 deletions were identified, of which more than half (56.4%, 74/131) were detected only once, in agreement with the high allelic heterogeneity described in the *DMD* gene. In contrast with findings in a previous paper [20], we observed a greater diversity at the distal hot spot (60 deletions) than at the proximal hot spot (46 deletions). Furthermore, deletions starting at the proximal hot spot had an average larger extension (14 deleted exons) than those starting at the distal hot spot (6 deleted exons). Deletions associated with BMD presented lower heterogeneity since the 10 most frequent deletions accounted for 76%. In contrast, in DMD, 12 recurrent deletions represented 46% of all deletions.

### Exonic duplications

Duplications accounted for 11.1% of all identified mutations. Unlike deletions, duplications had a more dispersed distribution and were slightly more frequent in the proximal hot spot (41.5%, 27/65) than in the distal hot spot (32.3%, 21/65) (Fig 2B). Like deletions, duplications presented a great heterogeneity because 36 of the 47 identified duplications were detected only once. Unlike deletions, duplications had more diversity in the proximal hot spot (25 duplications) than in the distal hotspot (13 duplications). Breakpoints were concentrated at the proximal hot spot, in intron 2 (10%, 13/130), intron 7 (7.7%, 10/130), intron 1 (7.7 / 10/130) and intron 9 (4.6%, 6/130). Distal breakpoints in duplications were less frequent than in deletions. Intron 7 presented similar breakpoints frequency between deletions and duplications with analogous proportions for 5' and 3' breakpoints.

No significant difference was observed in the size of the duplication between the proximal and distal hot spots. The most frequent duplication was that of exon 2, 7.7% (5/65), and it was associated with DMD in all cases. Duplications of exons 3–7, 5, 8–9, 50 and 56–62 were identified three times (4.6% all).

### Small mutations

**Fig 3:**
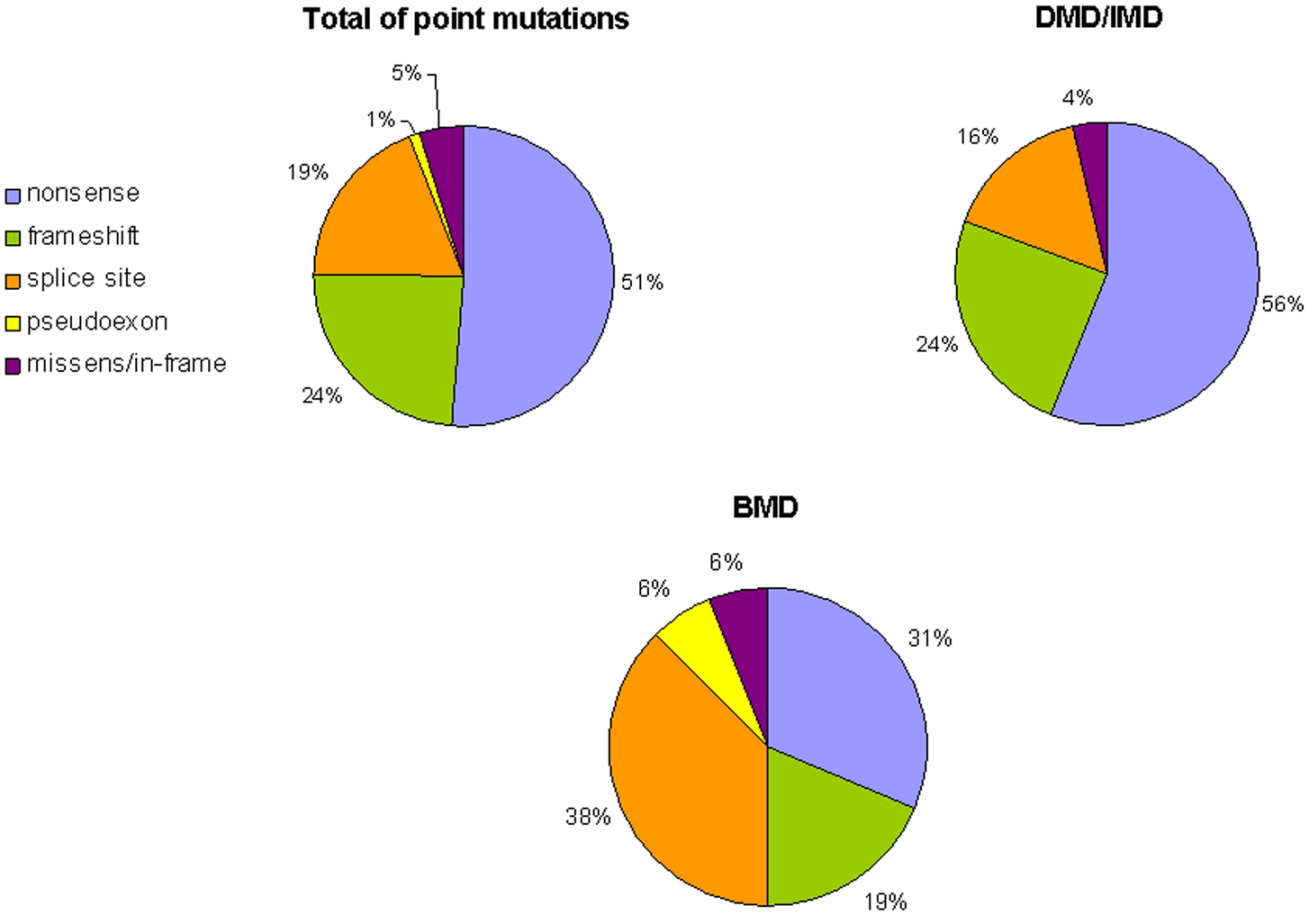
Distribution of identified point mutations and phenotypic groups.

**Fig 4:**
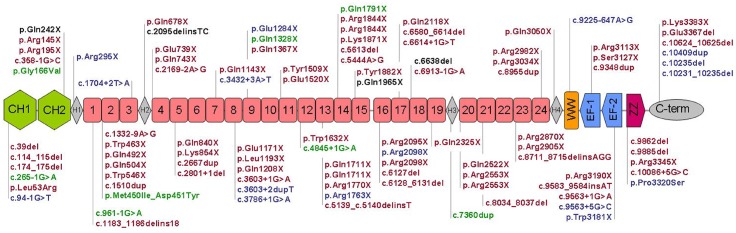
Distribution of point mutations distribution along the dystrophin domains. Mutations in DMD in red, in IMD in green, and in BMD in blue. Mutations detected in female isolated carriers in black. CH1-2: *calponin homology* domains binding actine ABD1; H1–H4: *hinge regions*; R1-24: spectrin-like repeats; WW: domain containing two tryptophans; EF-1-2: putative calcium binding sites; ZZ: *zinc-finger* domain.

**Fig 5:**
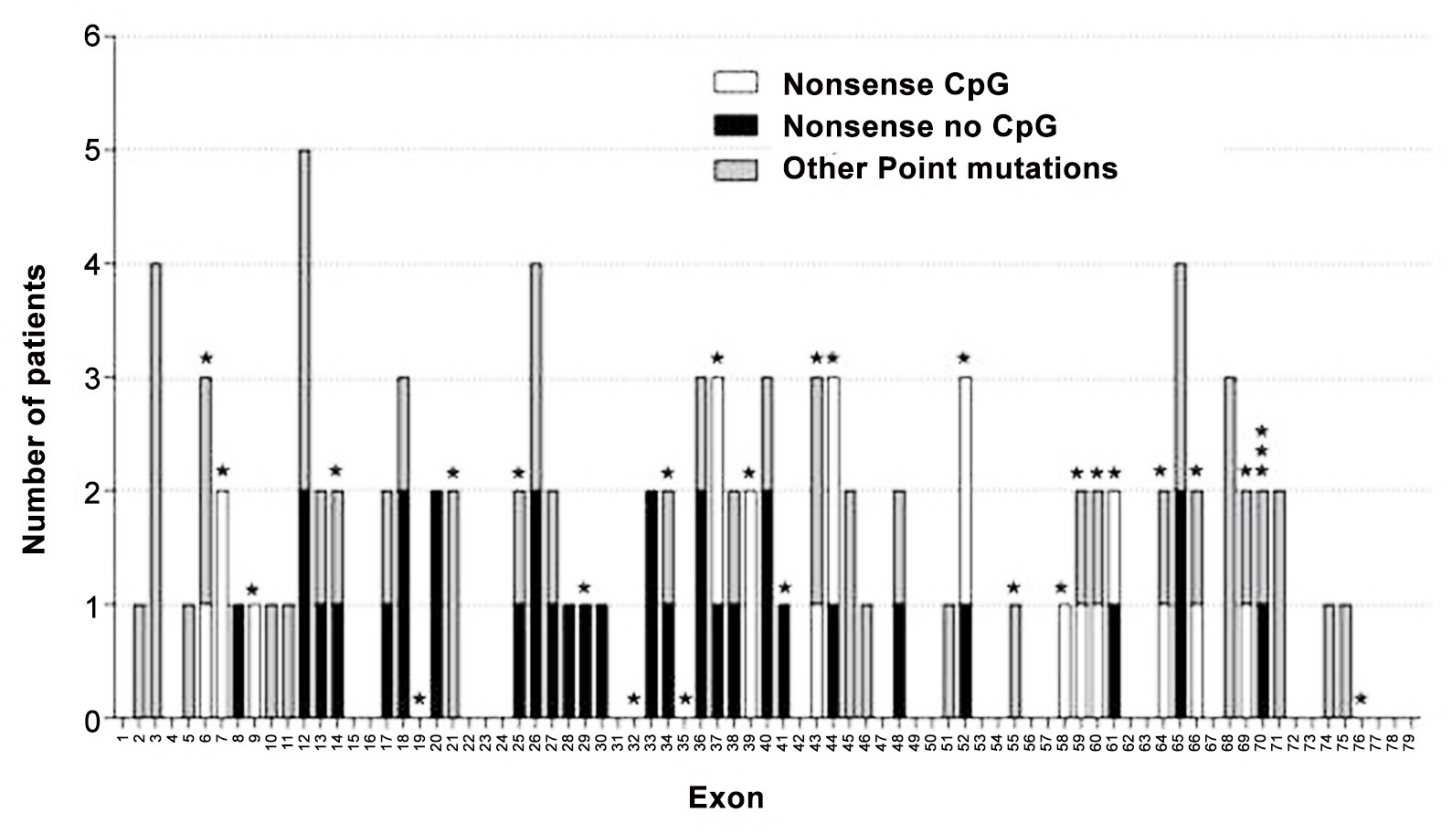
Distribution of point mutations identified at *DMD* exons. Asterisks indicate exons containing CpG codons.

We identified 98 mutations in 105 unrelated individuals (18.2%, 105/576), 55 of which have not been previously identified in the LOVD database. We identified a variety of mutations: 54 nonsense mutations, 15 microdeletions, 11 insertions/duplications, 20 splice site mutations, 4 missense, and one deep intronic mutation. The frequency of mutation types differed according to the clinical phenotype (Fig 3). The vast majority of DMD patients presented frameshift or nonsense mutations, causing a premature stop codon and a truncated protein rapidly degraded by the NMDA process. (81%). Nonsense substitutions are the most common type in patients with DMD/IMD (56.1%). However, most mutations in BMD patients affected mRNA splicing process (43.8%), while nonsense and frameshift mutations accounted for 31.25% and 18.75%, respectively. No recurrent mutations or hot spots were identified because they are distributed throughout all *DMD* exons involving all dystrophin domains (Figs 4 and 5). Table 2 specifies the rate and mutational target size for each type of small mutation. Single base substitutions were 2.4 times more frequent than small deletions or insertions, presenting a 15.4 times higher rate. The mutational rates per nucleotide 6.02x10-9 (all single nucleotide substitutions) and 0.39x10-9 (insertions/deletions small) were slightly lower than those published in a previous study [18].

**Table 2:** Mutation rate per nuclotide and type of mutation. Mutational type target was calculated in nucleotides according to muscular isoform Dp427m (NM_004006). μ_x_ is the mutational rate per nucleotide and per generation for each mutation type.

Mutation type	Number of mutations	Target (nt)	μ_x_ mutational rate (x10^-9^)
**One base substitution**	66	1812	6.02
Substitution at splice sites	12	312	6.36
Nonsense substitution	54	1500	5.95
Transition at CpG sites	20	29	114.03
Transversion at CpG sites	0	7	0.00
Transition at not CpG sites	22	380	9.57
Transversion at not CpG sites	12	1091	1.82
**Small indels**	27	11370	0.39
Indels at CDS	26	11058	0.39
Indels at *splice sites*	1	312	0.53

### Nonsense mutations

Nonsense mutations represented 51.4% (54/105) of the small mutations and 9.4% (54/576) of the total mutations. The UGA stop codon was the most frequent (42.6%), followed by UAG (29.6%) and UAA (27.8%). The *DMD* gene (Dp427m muscle isoform) has a total of 1,500 triplets that can be mutated into stop codon by a substitution of a single nucleotide. Transitions (77.8%) were more frequent than transversions (22.2%), and the C> T transition was the most common (90.5%).

### Frameshift mutations

Frameshift mutations were identified in 23.8% (25/105) of patients with small mutations. They were caused by deletion, insertion or deletion-insertion events (generally involving 1–5 nucleotides). Only two exceptions were observed: a deletion of 35 bp in exon 45 and an insertion of 18 bp in exon 11.

Most identified mutations in our cohort have not been previously described. Only two positions were recurrent although the mutations located therein were not identical: c.6127del and c.6128_6131del in exon 43 and c.10231_10235del and c.10235del in exon 71.

### Splicing mutations

**Fig 6:**
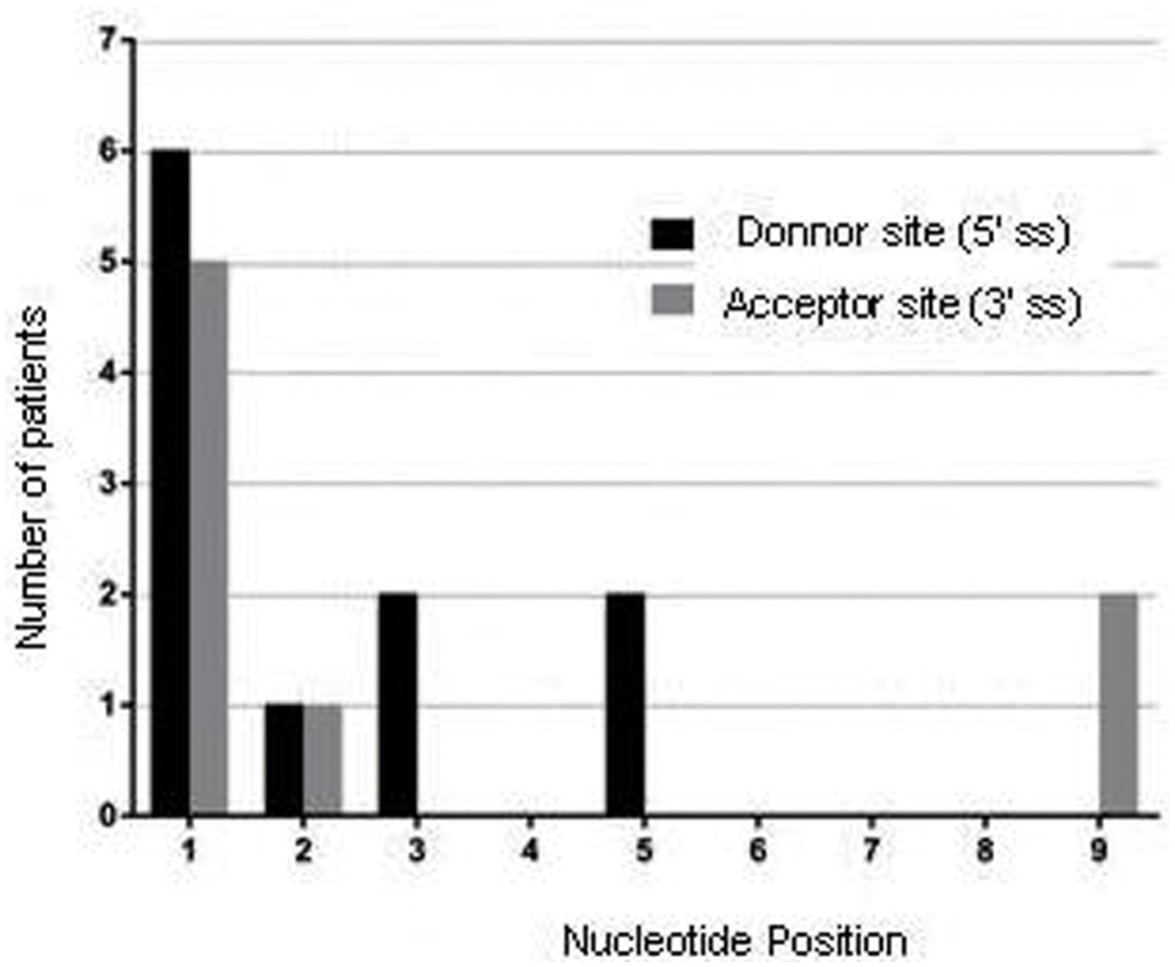
Distribution of the splice site mutations according to exon/intron boundaries. Nucleotide positions in x axis. Donor sites (in black) from +1 to +9 positions. Acceptor site (in grey) from -1 to -9 position. Number of mutations identified in each position in y axis.

Twenty splice site mutations were identified in 21 unrelated patients, representing 20% (21/105) of small mutations and 3.6% (21/576) of total mutations. Most of these changes were located in the canonical dinucleotides AG/GT of natural splice sites (65%, 13/20); while mutations located in less conserved positions were rare (Fig 6). Mutations affecting donor sites (5' ss) were slightly more frequent (52.4%, 11/21) than those affecting acceptor sites (3' ss) (38.1% 21/08). Two mutations were located outside natural splice sites. The deep intronic mutation C.9225-647A located in intron 62 was detected through muscular cDNA analysis. This mutation activated a cryptic donor site that, along with a cryptic acceptor, caused the inclusion of a 67 bp pseudoexon at the mature mRNA level. The second mutation was an apparent missense mutation at exon 38, c.5444A> G/ p.Asp1815Gly. The cDNA analysis showed that the mutation caused a 5 bp deletion due to the creation of a new donor site. The splicing pattern of 14 splice site mutations was determined. In most cases (78.6%, 11/14) splicing mutations caused complex patterns with levels of more than one transcript [19].

### Missense and in-frame mutations

Only four missense mutations and one in-frame deletion of a single amino acid (4.8% of point mutations and 0.9% of total mutations) were identified. Four of these mutations were associated with DMD and IMD phenotypes, while only one was associated with an atypical BMD patient. Two of the four were located at the N-terminal actin binding domain (ABD1): p.Leu53Arg in a DMD patient with irregular dystrophin reduction and p.Gly166Val in an IMD patient without muscle biopsy. A double aminoacid change, p.Met450Ile_Asp451Tyr, located at R2 of central rod domain, was identified in an IMD patient with irregular dystrophin reduction. Two mutations were identified in the distal domains. The p.Pro3320Ser mutation, located in ZZ domain (zinc-finger) of the cystein rich region (CRD), was identified in a BMD patient with near normal dystrophin levels. This patient presented an atypical phenotype with microphthalmia in the right eye, vitreous hyperplasia and severe cognitive delay. The deletion p.Glu3367del located in the C-terminal domain was identified in a DMD patient without immunohistochemistry data.

The small mutations identified in this work will be submitted to LOVD database.

## Discussion

This is the third largest series of dystrophinopathy patients described to date, with 576 unrelated patients [18, 20, 21]. The results illustrate the high allelic heterogeneity of the *DMD* gene. We identified 276 different mutations, 85 of which have not been previously described. One hundred and thirty-one were exonic deletions, 46 were exonic duplications, one was a complex rearrangement deletion/duplication, and 98 were small mutations.

The diagnostic algorithm used consisted of the analysis of the exonic copy number, followed by the analysis of small mutations by sequencing genomic DNA or cDNA from muscle biopsy when available, for negative exonic deletions/duplications. Our results confirm that it is an accurate algorithm for the molecular diagnosis of dystrophynopathies. Despite the study of muscle cDNA requires an invasive method as muscle biopsy, it allows: 1) detection of all types of mutations, including deep intronic mutations, 2) assessment of the impact of DNA variants on pre-mRNA splicing to identify splice sites (ss) abnormalities or regulatory elements (SRE), and 3) evaluation of the reading frame conservation that determines the phenotypic severity. An example is the mutation c.5444A> G, which could have been classified as a non-pathogenic missense change (p.Asp1815Gly) when in fact it caused aberrant splicing, generating a frameshift transcript. However, care must be taken in the study of point mutations in mRNA; some splicing mutations in BMD patients can induce significant levels of normal transcripts, making it difficult to detect aberrant mRNA variants. This is the case of the mutation c.3603 + 2dupT in which the anomalous transcript only represented 17% of total transcripts.

The frequency for each mutation type was similar to that described previously [22] and differed between DMD and BMD phenotypes (Figs 1 and 3). BMD patients had fewer point mutations and more exonic deletions than DMD patients. These differences were more pronounced in the point mutations. In DMD patients, we found most point mutations were frameshift or nonsense type, according to the reading frame. However, our BMD patients had a high frequency of splicing mutations, although they presented many exceptions to the reading frame law, as up to 50% of mutations were frameshift or nonsense type.

In accordance with previous studies [18, 20, 23, 24], we noted that large deletions and duplications had a non-random distribution with the presence of two hot spots. In our cohort, as in a previous study [25], the intronic size was not the only factor determining deletional profiles. Several mechanisms involved in the development of genomic rearrangements have been reported [26–28]. The non-allelic homologous recombination (NAHR, non-allelic homologous recombination) is a mechanism described in numerous genetic disorders that can cause deletions and duplications in the same frequency [29–31]. The *Alu* repeats crosslinking by the NAHR mechanism have been considered a cause of deletions in several genes, including the *DMD* gene [32]. Analysis of breakpoints in the *DMD* gene shows that exonic deletions and duplications present different frequencies, caused by different mechanisms. If both, deletions and duplications were the result of NAHR, we would expect similar deletion and duplication frequencies for each intron. It has also been suggested that duplications could arise at different times in the cellular cycle. Like point mutations, the deletions are predominantly maternally inherited, while duplications descend from the paternal germ line [26,33,34]. It has been reported that the duplication of exon 2 is caused by the non-homologous end joining mechanism (NHEJ) involved in the double strand DNA break repair [24].

Unlike deletions/duplications, point mutations have no hot spots and are distributed along the entire gene (Fig 4). The only exceptions are missense mutations that mainly concentrate in the N- and C-terminal ends of binding protein domains, in agreement with previous publications [20]. Half of the identified mutations in our study were not previously described, confirming the necessity to study the whole gene in molecular diagnosis.

### Genotype/phenotype correlation

The phenotypic severity depends mainly on the reading frame rule [4]. This rule postulates that mutations destroying the reading frame cause absence of dystrophin in skeletal muscle and the DMD phenotype, whereas mutations preserving the reading frame permit the expression of semifunctional dystrophin and BMD phenotype. The workflow (MLPA, sequencing, *in silico* analysis and mRNA analysis) used in this study has allowed us to better find exceptions to the reading frame rule and to better understand the genotype-phenotype correlation. In our cohort, the reading frame rule was fulfilled in 90.4% of DMD patients and in 82.4% of BMD patients. Exonic deletions were the mutational group that had fewest exceptions to the rule (8.4%, 32/379), while exonic duplications and point mutations presented exceptions in similar proportions, 23.3% (14/60) and 19.2% (19/99) respectively.

**Fig 7:**
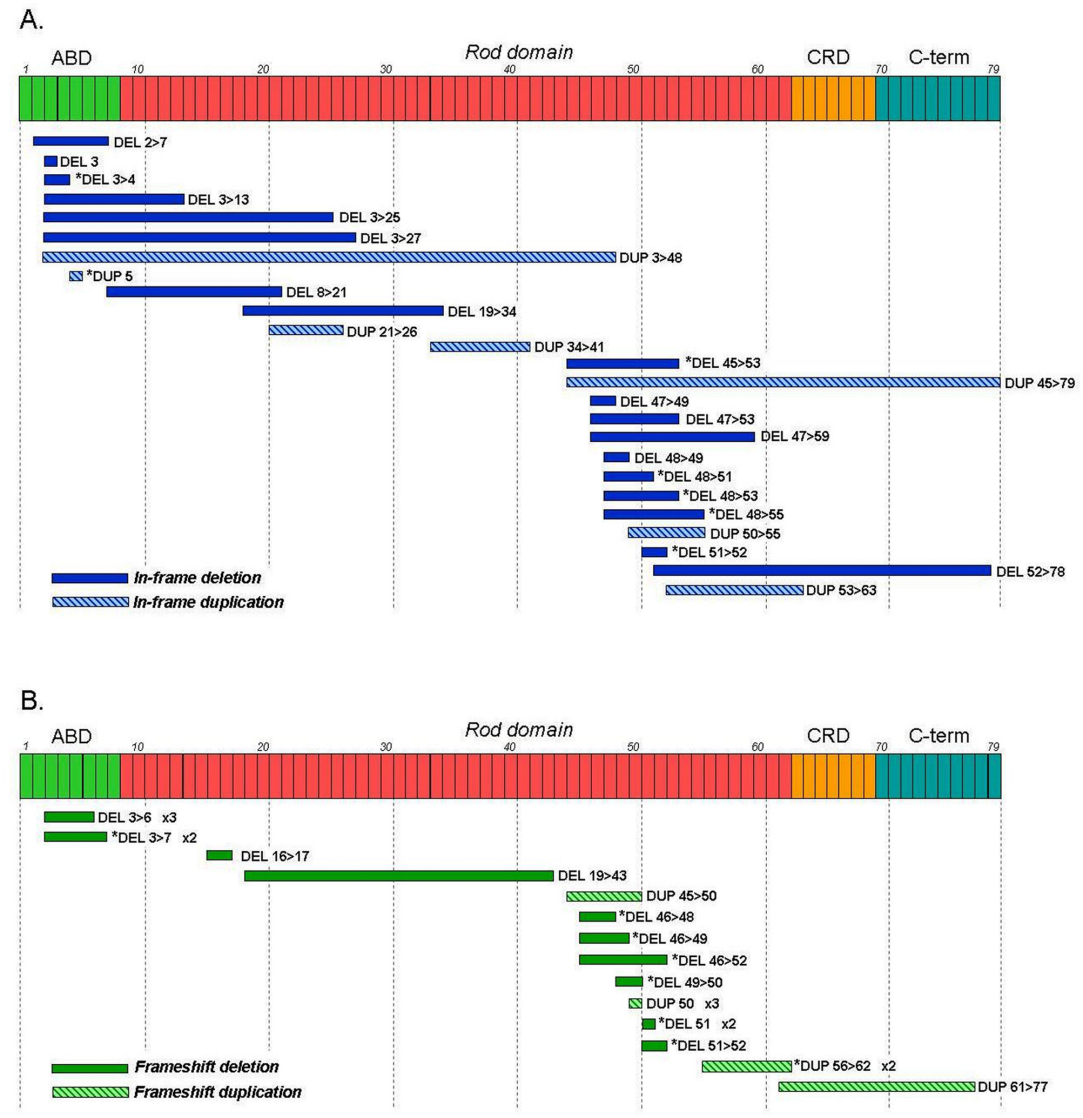
Exceptions to the reading frame rule in large intragenic rearrangements. (A) In-frame mutations in DMD patients. (B) Frameshift mutations in BMD patients. Asterisk indicates mutations identified in both phenotypes.

Several mechanisms explaining the exceptions to the reading frame law have been described. It is assumed that duplications occur in tandem, without having an mRNA study to support this in most cases. It seems certain that most are tandem duplications [35] because noncontiguous rearrangements cases have been described causing complex effects at the mRNA level [24]. Furthermore, it is unknown how large in-frame duplications can affect the dystrophin function. Regarding deletions several considerations can be made. In DMD patients, 44% (8/18) of the identified exceptions were deletions causing partial or total loss of a binding protein domain that would compromise the protein function. In 7 patients, the mutation affected the ABD domain and in one patient it affected the CRD region (Fig 7). Deletions affecting the brain and muscle promoters were associated in all cases with the DMD phenotype. According to previous descriptions, the two frameshift deletions at the 5’ end (3>6 and 3>7) accounted for 36% of the exceptions associated with BMD. Several mechanisms seem to be involved in the rescue of dystrophin expression in frameshift mutations located at the 5' end. These mechanisms include alternative translation initiation in exon 6 or 8 [36,37] and patterns of alternative splicing [38].

Small mutations also present numerous exceptions to the reading frame rule, especially in BMD patients, in whom most exceptions are due to alterations in the normal splicing pattern [39]. All nonsense or frameshift mutations associated with BMD are located in in-frame exons (exons 9, 28, 37, 71 and 74). In these mutations, the alteration of splicing regulatory elements (SRE) can increase the levels of transcripts with in-frame skipping, leading to the rescue of the phenotype [40]. In severe DMD or IMD patients, all exceptions are missense mutations located in critical domains for protein function. Previous publications [18] assumed that mutations in natural splice sites always cause exon-skipping. mRNA analysis shows that these mutations often cause complex splicing patterns which may include skipping of one or more exons, activation of cryptic splice sites and intronic retention.

Concerning altered splicing patterns, two types of *Alu* retrotransposon insertions have been described in the *DMD* gene: one in an XLCM patient, and the other in a DMD patient [41,42]. In both cases, the insertion of the *Alu* element occurred inside the intron, modifying the splicing pattern. This altered pattern caused the skipping of a closer exon in one case and the insertion of the *Alu* element in the mature mRNA in the other case. Patient #1990 presented an insertion of 18 bp in exon 11. This inserted fragment showed a high sequence homology with part of the retrotransposon elements of the *Alu* J family. In our patient, we were unable to determine whether the insertion altered the splicing because no muscle biopsy was available for mRNA analysis.

### Protein domains changes: missense and in-frame mutations

Missense mutations are exceptions to the reading frame rule and often cause an unusual combination of severe phenotypes with dystrophin expression. According to previous publications [18, 20], missense mutations are rare (0.9%) and most are located in protein-protein interaction domains. However, missense DMD mutations have proven to be valuable in the analysis of pathological dystrophin alterations.

In our cohort, we identified two mutations in the N-terminal F-actin binding domain (ABD1). This domain is composed of two calponin homologous (CH1-2) modules with three actin-binding regions (ABS1-3). The domain ABD1, along with an additional domain located at the rod domain (ABD2), allows dystrophin to combine with actin filaments, forming bundles and networks and avoiding the depolymerization of filaments [43] (Fig 4). It has been reported that missense mutations in the ABD1 domain decrease the thermodynamic stability of dystrophin, causing misfolding and aggregation into amyloid-like structures [44]. We identified the mutation p.Leu53Arg in the CH1 module in a DMD patient, whereas the mutation p.Gly166Val in the CH2 module was identified in an IMD patient. The difference in clinical severity between the two mutations suggests they affect the structure of ABD1 domain and its ability to interact with actin differently. The domain ABD1 was the first protein domain in which the crystal structure was determined (S2A Fig) [45]. The p.Leu53Arg mutation in CH1 introduces a charged residue on a hydrophobic region that is structurally closer to the actin binding sites (ABS1-3) (S2B Fig). Hypothetically, the mutation could compromise the binding with actin and cause a DMD phenotype even without altering the dystrophin expression. According to this hypothesis, a mutation in the adjacent residue (p.Leu54Arg) has been described in a DMD patient [46]. However, the mutation in CH2 (p.Gly166Val) is not structurally located close to ABS3, indicating it does not interact with actin. This mutation replaces a small neutral residue with a hydrophobic residue at the beginning of an alpha helix where mutations associated with BMD have been described (Asp165, Ala168 and Ala171) [47]. This suggests that this region is critical for CH2 stability (S2C Fig). Our data concerning the location of the mutated residue in relation to phenotype correlate with functional studies by Henderson and co-workers [48]. These authors suggested that missense mutations located in CH1 are associated with severe phenotype due to the combined effect of alterations in actin binding and induction of thermodynamic instability. They observed that p.Leu54Arg decreases the actin binding affinity four-fold and induces aggregation and thermal denaturation. In contrast, p.Asp165Val, p.Ala168Asp and other mutations located in CH2, associated with BMD, increase protein aggregation and denaturation without altering the actin binding affinity [48].

In our cohort, a mutation consisting in two amino acid substitutions in the central rod domain (p.Met450Ile_Asp451Tyr) was identified in IMD patient #1215. To our knowledge, several missense mutations have been described in this domain in the literature [49,50] and in LOVD. The rod domain consists of 24 repeats similar to spectrin (R1–R24) and 4 hinge regions (H1–H4) [51] (Fig 4). It is believed that the dystrophin acts by protecting the membrane from damage caused by muscle contraction. Spectrin-like repeats participate in this biomechanical function, acting as springy units that can be deployed when they are subjected to strength [52]. Initially, it was thought that the spectrin-like repeats had a very low importance in the overall function of dystrophin, since in-frame deletions removing large portions of the rod domain were observed in mild phenotypes [53]. However, several studies suggest that the rod domain has a more complex role than a purely mechanical function. The study of therapeutic mini-dystrophin in *mdx* mice revealed that 3 or more repeats are required to retain the protein functionality [54, 55]. Moreover, several repeats interact with ligands such as F-actin, sinemin, Par-1, nNOS and phospholipids [56–60].

Although the repeats of the spectrin protein family (spectrin, dystrophin, utrophin, α-actinin, etc) exhibit a relatively low homology they present a heptade periodic pattern that is characteristic of coiled-coil structures (S3A Fig). This pattern consists of seven hydrophobic and hydrophilic residues represented by the letters *a to g*. The “*a*” and “*d*” residues are mostly hydrophobic and ensure the antiparallel folding of three alpha helices (HA, HB and HC) forming a supercoiled bundle or coiled-coil [61]. The other positions are usually occupied by hydrophilic or charged residues, so that the helix is coiled between them, hiding the hydrophobic residues inside the bundle and exposing the hydrophilic/charged residues outside the bundle (S3B Fig). It is believed that dystrophin adjacent repeats are joined by a connector that ensures the helicoidal continuity of the last helix in the first repeat (HC) with the first helix in the next repeat (HA) [62]. The double change p.Met450Ile_Asp451Tyr identified in IMD patient #1215 is located at the beginning of the HA helix of the R2 repeat (S3C Fig). Dystrophin immunostaining shows dystrophin and presence of negative fibers since the change induces instability or folding defects leading to dystrophin degradation. According to this hypothesis, the alignment of repeats indicates that the mutated residues are located in heptad positions “*a*” (Met450) and *"b"* (Aps451), suggesting that the mutation alters the coiled coil structure of R2 repeat (S3A Fig).

Several algorithms have been described to predict coiled coil domains. One of these algorithms, MARCOIL, establishes the tendency to form coiled coils in a certain heptade position for each residue [63]. The MARCOIL analysis of the wild-type dystrophin and p.Met450Ile_Asp451Tyr dystrophin shows that the mutation causes a dramatic change in the probability of forming coiled coils of 434–477 residues, corresponding to HC helix of R1 and HA helix of R2 (S3D Fig). These data suggest that the mutation changes the protein microenvironment of R1–R2 leading to misfolding and/or thermodynamic instability of the protein. Recently it has been described the presence of p.Leu427Pro mutation in R1 repeat in two brothers with mild BMD phenotype. The patients have a weak immunostaining in N-terminal region of dystrophin (ABD1 and H1) and the mutation would originate a partial misfolding. These authors suggested that the mutation affects the dynamic properties of the entire N-terminal region [49]. Moreover, it has been described that the combination of two polymorphic variants (p.Glu2910Val and p.Asn2912Asp) in the R23 repeat causes DMD due to the destabilization of repeat [50]. These changes, along with the mutation identified in our patient, correlate with mini-dystrophin studies [54] and a BMD patient who presented minimal muscle involvement and in-frame deletion of exons 14 to 48 (R3–R19) [53]. These data suggest that regions R1-3 and R20-24 are essential for the proper function of dystrophin. Some authors have hypothesized that these regions contribute to the stabilization of the adjacent N- and C-terminus domains involved in protein-protein interactions [49,50].

In the cysteine-rich region, the mutation p.Pro3320Ser at ZZ domain (zinc-finger domain) (Fig 4) was identified in a patient with atypical BMD phenotype. This patient, #1497, presented mild muscle weakness, microophthalmia,vitreous hyperplasia, severe cognitive delay and, interestingly, almost normal dystrophin expression in skeletal muscle. ZZ domains are small protein motifs that have up to four conserved cysteines allowing zinc binding or other metals (S4A and S4B Fig) [64]. The zinc binding induces conformational changes permitting binding to specific ligands, such as DNA or other proteins [65]. The cysteine rich region of dystrophin (CRD) has been implicated in binding to β-dystroglycan [66–68]. The CRD region comprises several modular domains: a WW domain, two putative calcium binding sites (EF-hand 1 and 2) and one ZZ domain. The WW domain contains two conserved tryptophans (W) that allows the binding of dystrophin with the last 15 C-terminal residues of β-dystroglycan (S4C Fig). Some studies demonstrate that WW domain is not sufficient by itself to establish the union with β-dystroglycan and requires the presence of both EF and ZZ domains for a stable interaction [69–71]. In 2007, Hnia and co-workers identified two zinc-binding regions and one point interacting to β-dystroglycan in the ZZ domain (S4B Fig) [70]. The p.Pro3320Ser mutation, identified in patient #1497, is located in the first zinc-binding region, adjacent to the Cys3319 residue (S4B Fig). This location suggests that the change alters the ability to bind zinc in the first region and consequently alters the conformation of the ZZ domain involving the interaction with β-dystroglycan. The BMD phenotype indicates that the mutation does not completely destroy interaction with β-dystroglycan, as expected by a severe DMD phenotype. We observed that mutations located in the first zinc-binding region are mainly associated with the BMD phenotype, whereas those located in the second zinc-binding region, which includes the anchor point to β-dystroglycan, are associated with the DMD phenotype (S4B Fig). The patient’s severe mental retardation correlates with the location of the mutation in the C-terminal end of the gene, affecting all *DMD* isoforms including Dp71. Dp71 is the most abundant form in fetal and adult brain [72] and has been described as the main factor determining the severity of cognitive impairment. Mutations in Dp71 are associated with severe mental retardation [73, 74]. The atypical clinical presentation of the patient, microphthalmia and vitreous hyperplasia which are not associated with dystrophynopathy, encompass some forms of congenital muscular dystrophy caused by glycosylation defects of α-dystroglycan. The fact that the mutation alters the interaction with β-dystroglycan questions whether the structural changes are caused by dystrophinopathy or whether there are two separate pathologies.

In the C-terminal domain, we identified a deletion of a single amino acid (p.Glu3367del) associated with the DMD phenotype. The mutation is located 13 residues from the ZZ domain in a region with unknown function, although it is one of the most conserved dystrophin regions. This mutation has been previously described in two IMD and one DMD patients. Becker and co-workers suggested that the deletion of the Glu3367 residue causes a torque and/or translation of an α-helix that may induce alterations in the conformation or function of the ZZ domain [75].

### Variability of *DMD* gene

X-linked diseases provide data on the human genome mutation rate [76,77]. Direct and indirect measures suggest that the genomic mutation rate is 1-2x10^-8^ per nucleotide. The rate per nucleotide mutation observed in our sample was 0.6x10^-8^, slightly lower than described to date. This could be due to enrichment with large rearrangements respect to point mutations in our sample. According to spontaneous deamination of methylated cytosines [78], CpG transitions in the CGA codon (arginine) represent 37% of total nonsense identified mutations although this odon represents only 2% of the target codons. The 11.9-fold difference in the number of CpG transitions compared to non-CpG transitions is similar to that obtained by measuring the divergence between humans and chimpanzees, observed to be 10 times higher for CpG sites [79]. According to previous publications [39], there are no hot spots for small insertions/deletions, although the study of the mutated sequence reveals that in most cases the presence of repeated motifs is implicated in the etiology of such mutations.

The sequencing of the coding and flanking intron regions allowed us to identify 119 SNPs (S1 and S2 Tables), 21 of which had not been previously described in LOVD database, dbSNP and HapMap. The study of haplotypes obtained by sequencing reveals the variability in the *DMD* gene. Just as the point mutations study reveals that 56% are private mutations, study of the SNPs shows that the vast majority of unrelated families present a unique haplotype for *DMD* locus. This probably reflects the high rate of *DMD* recombination linked to its large size. Of the 119 identified SNPs, 93 are located in intron regions and 26 in coding exons. Of these 26, 11 were synonymous and 15 were non-synonymous changes, involving the substitution of one amino acid for another. Two non-synonymous SNPs, p.Lys1491Thr and p.Met1483Val, have not been described previously. Both are located in exon 32, corresponding to the R11 repeat of rod domain. However, we can not affirm whether they are rare variants or whether they have a pathogenic effect.

## Conclusions

The diagnostic algorithm described in this work was accurate for the molecular diagnosis of dystrophinopathies. It analyzed the exonic copy number and point mutations by sequencing cDNA from muscle biopsy or genomic DNA for negative deletions/duplications. There were no hot spots for small insertions/deletions. The study of the mutated sequences showed that repeated motifs were implied in the etiology in most cases. A detailed analysis of missense mutations contributed significantly to understanding their impact on the protein structure and to explain the non-fulfilment of the reading frame law regarding genotype/phenotype correlation.

## Supporting Information

S1 Fig Complex rearrangement in #1612 DMD patient: del Pb>29 and dup 37>43. (A) MLPA results. (B) Diagram of DMD mutated gene in this patient.
(TIF)

S2 Fig Missense mutations in N-terminal acting binding domain (N-ABD1). (A) Tridimensional structure of N-ABD domain with calponin-like domains (CH 1–2). ABS1: residues from 17 to 26; ABS2: residues from 102–114; ABS3: residues from 130–146. (B) Module CH1 with the Leu54 residue that reduces four times the actin binding affinity, (C) Module CH2: mutations associated to BMD phenotype leading to thermodynamic instability and not altering actin binding.
(TIF)

S3 Fig Double missense mutation at R2 in rod domain and its impact on dystrophin. a. R1 and R2 tridimensional structure: mutated residues and the three helix HA,HB and HC show antiparallel coiled-coil formation. b. HA alienation with seven spectin-like repeats from other proteins. Residues with sequence homology are coloured. Heptade pattern with hydrophobic residues at “a” and “d” positions. c. Diagram showing a trimeric coiled-coil: hydrophobic residues at the nucleus and hydrophilic/loaded residues outside. d. MARCOIL result for coiled coils in residues 420–527: wild type protein (in blue) and mutated p.Met450Ile_Asp451Tyr protein (in red).
(TIF)

S4 Fig Missense mutation at ZZ domain. (A) Murine CBP ZZ domain residues coordinating zinc binding in yellow. (B) Diagram of dystrophin and β-dystroglican binding. (C) Dystrophin ZZ domain diagram showing the two zinc-binding regions. Zn-co-ordinating residues In yellow; Pro3320 mutated residue in red and the second interaction point site with β-dystroglican in orange. Arrows indicate missense mutations described in *LOVD* database: DMD patients in black and BMD patients in blue.
(TIF)

S1 Table SNPs in coding region. *Asterisks indicate previously nondescribed changes with unknown pathogenic effect.
(DOC)

S2 Table SNPs in non-coding regions. *Asterisks indicate previously nondescribed changes with unknown pathogenic effect.
(DOC)
