# Supplementary figures and images for: DMD Mutations in 576 Dystrophinopathy Families: A Step Forward in Genotype-Phenotype Correlations
Source: PLoS One. 2015 Aug 18;10(8):e0135189. doi: 10.1371/journal.pone.0135189 (PMC4540588; doi:10.1371/journal.pone.0135189)

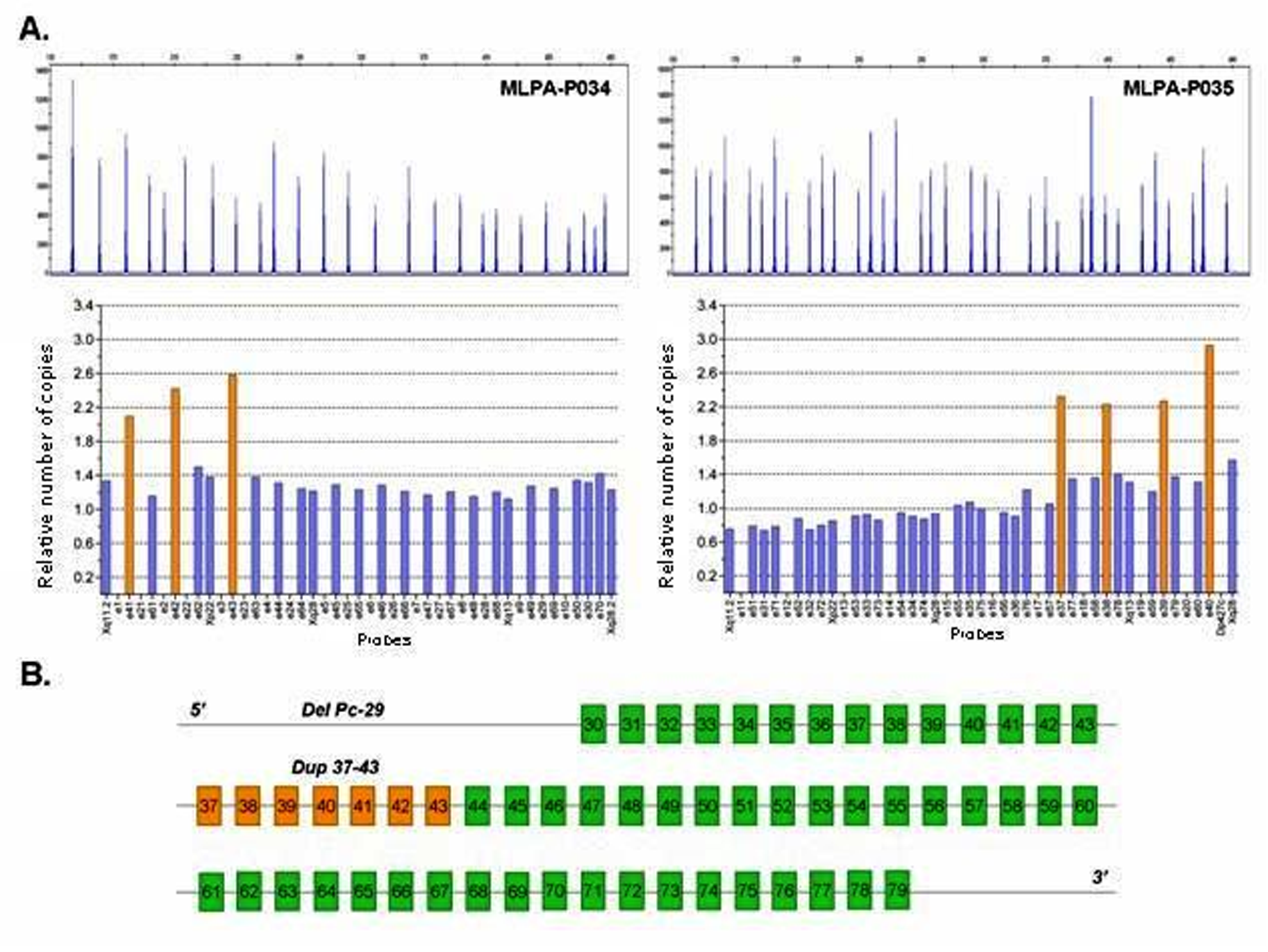

Supplement: S1 Fig — (A) MLPA results. (B) Diagram of DMD mutated gene in this patient. (TIF) [file pone.0135189.s001.tif]

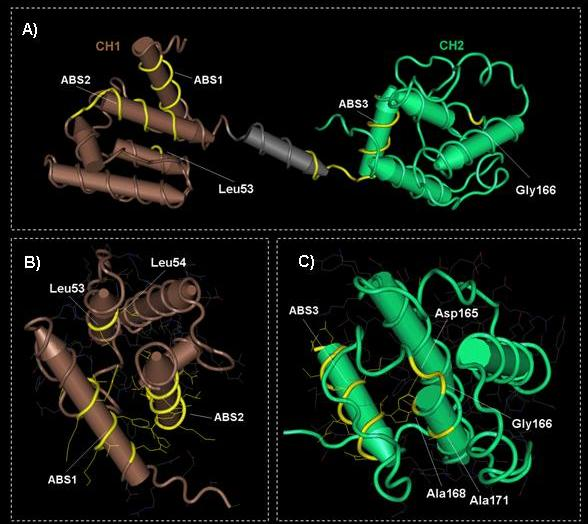

Supplement: S2 Fig — (A) Tridimensional structure of N-ABD domain with calponin-like domains (CH 1–2). ABS1: residues from 17 to 26; ABS2: residues from 102–114; ABS3: residues from 130–146. (B) Module CH1 with the Leu54 residue that reduces four times the actin binding affinity, (C) Module CH2: mutations associated to BMD phenotype leading to thermodynamic instability and not altering actin binding. (TIF) [file pone.0135189.s002.TIF]

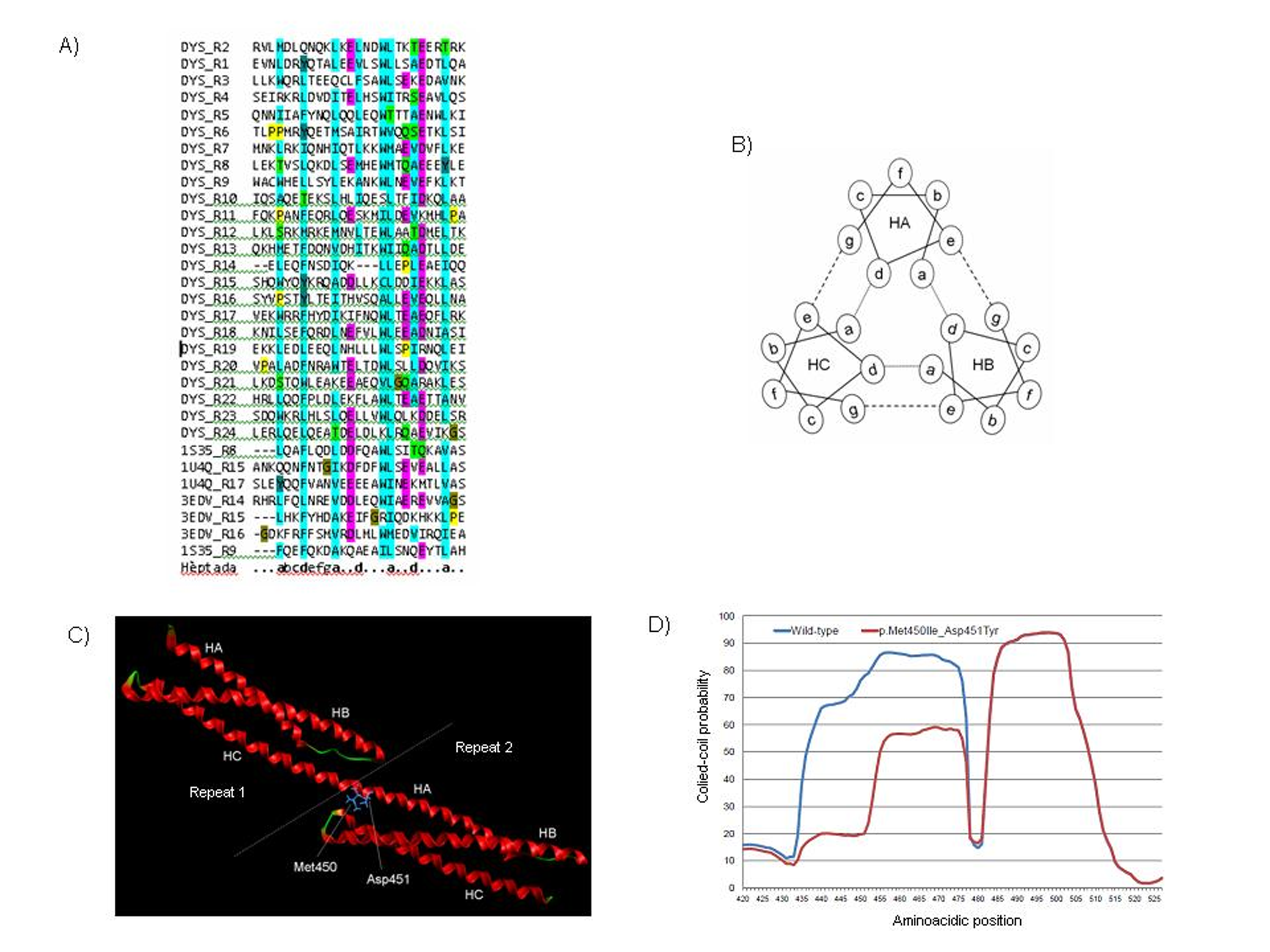

Supplement: S3 Fig — a. R1 and R2 tridimensional structure: mutated residues and the three helix HA,HB and HC show antiparallel coiled-coil formation. b. HA alienation with seven spectin-like repeats from other proteins. Residues with sequence homology are coloured. Heptade pattern with hydrophobic residues at “a” and “d” positions. c. Diagram showing a trimeric coiled-coil: hydrophobic residues at the nucleus and hydrophilic/loaded residues outside. d. MARCOIL result for coiled coils in residues 420–527: wild type protein (in blue) and mutated p.Met450Ile_Asp451Tyr protein (in red). (TIF) [file pone.0135189.s003.tif]

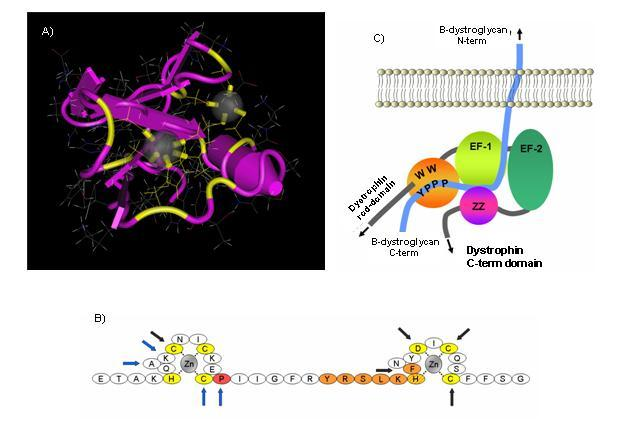

Supplement: S4 Fig — (A) Murine CBP ZZ domain residues coordinating zinc binding in yellow. (B) Diagram of dystrophin and β-dystroglican binding. (C) Dystrophin ZZ domain diagram showing the two zinc-binding regions. Zn-co-ordinating residues In yellow; Pro3320 mutated residue in red and the second interaction point site with β-dystroglican in orange. Arrows indicate missense mutations described in LOVD database: DMD patients in black and BMD patients in blue. (TIF) [file pone.0135189.s004.TIF]
