# Supplementary material for: DMD Mutations in 576 Dystrophinopathy Families: A Step Forward in Genotype-Phenotype Correlations
Source: PLoS One. 2015 Aug 18;10(8):e0135189. doi: 10.1371/journal.pone.0135189 (PMC4540588; doi:10.1371/journal.pone.0135189)
Supplement: S1 Table — *Asterisks indicate previously nondescribed changes with unknown pathogenic effect. (DOC) [file pone.0135189.s005.doc]

**Aditional file 6: Table S1**. **SNPs in coding region.** *Asterisks indicate previously nondescribed changes with unknown pathogenic effect.

| **Exon** | **DNA** | **Protein** | **Frequency** |
| --- | --- | --- | --- |
| 3 | c.152T>A | p.= | 0.013 |
| 5 | *c.303T>C | p.= | 0.026 |
| 8 | c.802T>C | p.= | 0.013 |
| 9 | c.837G>A | p.= | 0.052 |
| 10 | c.1098A>T | p.= | 0.013 |
| 11 | c.1225A>T | p.Thr409Ser | 0.013 |
| 14 | c.1635A>G | p.= | 0.169 |
| 16 | c.1869C>T | p.= | 0.013 |
| 20 | c.2391T>G | p.Asn797Lys | 0.013 |
| 21 | c.2645G>A | p.Gly882Asp | 0.364 |
| 23 | c.3021G>A | p.= | 0.013 |
| 32 | *c.4472A>C | p.Lys1491Thr | 0.013 |
| 32 | *c.4447A>G | p.Met1483Val | 0.013 |
| 37 | c.5234G>A | p.Arg1745His | 0.506 |
| 39 | *c.5419C>T | p.= | 0.013 |
| 42 | c.6105G>T | p.Glu2035Asp | 0.143 |
| 43 | c.6143G>A | p.Ser2048Asn | 0.039 |
| 45 | c.6463C>T | p.Arg2155Trp | 0.078 |
| 48 | c.7096C>A | p.Gln2366Lys | 0.545 |
| 53 | c.7728T>C | p.= | 0.169 |
| 59 | c.8810A>G | p.Gln2937Arg | 0.195 |
| 59 | c.8762A>G | p.His2921Arg | 0.026 |
| 59 | c.8729A>T | p.Glu2910Val | 0.013 |
| 59 | c.8734A>G | p.Asn2912Asp | 0.013 |
| 59 | c.8767G>T | p.Ala2923Ser | 0.013 |
| 75 | c.10789C>T | p.= | 0.013 |
