# Supplementary material for: DMD Mutations in 576 Dystrophinopathy Families: A Step Forward in Genotype-Phenotype Correlations
Source: PLoS One. 2015 Aug 18;10(8):e0135189. doi: 10.1371/journal.pone.0135189 (PMC4540588; doi:10.1371/journal.pone.0135189)
Supplement: S2 Table — *Asterisks indicate previously nondescribed changes with unknown pathogenic effect. (DOC) [file pone.0135189.s006.doc]

**Aditional file 7: Table S2. SNPs in non-coding regions.** *Asterisks indicate previously nondescribed changes with unknown pathogenic effect.

| **Intron** | **DNA** | **Frequency** |
| --- | --- | --- |
| 2 | c.93+303T>A | 0.052 |
| 2 | c.94-9dupT | 0.013 |
| 2 | c.94-133G>A | 0.156 |
| 2 | c.94-17_94-16insT | 0.013 |
| 5 | c.358-139G>A | 0.013 |
| 5 | *c.357+170C>A | 0.013 |
| 7 | c.649+145T>C | 0.221 |
| 8 | c.831+148_831+149dup | 0.195 |
| 8 | c.960+50delG | 0.013 |
| 8 | *c.831+144_831+145del | 0.013 |
| 8 | c.832-54 A>G | 0.026 |
| 8 | *c.832-53C>T | 0.013 |
| 8 | c.832-18_832-17delinsGA | 0.013 |
| 9 | *c.960+166T>C | 0.013 |
| 9 | c.960+50del | 0.013 |
| 9 | c.961-109A>G | 0.039 |
| 9 | c.961-124G>C | 0.013 |
| 11 | c.1331+55A>G | 0.013 |
| 11 | c.1331+127G>A | 0.078 |
| 12 | c.1482+178 A>G | 0.013 |
| 12 | c.1483-110G>A | 0.091 |
| 12 | c.1483-123G>T | 0.065 |
| 12 | c.1483-67A>T | 0.026 |
| 14 | c.1704+51T>C | 0.065 |
| 14 | c.1812+118A>G | 0.013 |
| 15 | c.1993-37T>G | 0.013 |
| 15 | *c.1813-23T>C | 0.013 |
| 16 | c.2168+13T>C | 0.013 |
| 16 | *c.1992+102G>T | 0.013 |
| 16 | c.1993-37T>G | 0.429 |
| 17 | c.2168+13T>C | 0.195 |
| 17 | c.2169-96T>C | 0.013 |
| 18 | *c.2293-123A>G | 0.013 |
| 20 | c.2622+33A>G | 0.013 |
| 20 | c.2623-11C>G | 0.013 |
| 24 | c.3277-152A>G | 0.169 |
| 24 | c.3277-30C>T | 0.013 |
| 26 | c.3603+15dupA | 0.039 |
| 26 | c.3786+296C>T | 0.013 |
| 28 | c.3921+128C>T | 0.247 |
| 28 | *c.3921+39G>A | 0.013 |
| 29 | *c.4072-213C>A | 0.013 |
| 30 | c.4234-13A>G | 0.065 |
| 30 | *c.4234-31A>G | 0.013 |
| 33 | *c.4675-87A>C | 0.013 |
| 34 | c.4845+69G>A | 0.013 |

| 34 | c.4846-153G>A | 0.052 |
| --- | --- | --- |
| 35 | c.5025+103G>A | 0.403 |
| 35 | c.5026-63T>A | 0.013 |
| 35 | c.5026-93A>G | 0.026 |
| 38 | c.5448+169A>T | 0.104 |
| 38 | *c.5448+67G>A | 0.026 |
| 39 | c.5586+94_5586+95dup | 0.273 |
| 39 | c.5586+96_5586+97del | 0.052 |
| 40 | c.5740-67G>T | 0.104 |
| 40 | *c.5740-102G>T | 0.013 |
| 41 | c.5922+77_5922+78del | 0.338 |
| 41 | c.5923-179G>A | 0.013 |
| 42 | c.6118-63_6118-62dup | 0.039 |
| 43 | c.6290+27T>A | 0.052 |
| 43 | c.6291-115G>A | 0.091 |
| 43 | *c.6291-39 A>G | 0.013 |
| 43 | *c.6291-155G>A | 0.013 |
| 45 | c.6614+26G>T | 0.325 |
| 45 | c.6615-27A>T | 0.013 |
| 46 | c.6762+141C>T | 0.013 |
| 47 | c.6912+124_6912+128dup | 0.026 |
| 47 | c.6913-114A>T | 0.052 |
| 49 | c.7200+53C>G | 0.195 |
| 50 | c.7309+176T>C | 0.026 |
| 50 | *c.7309+26delC | 0.078 |
| 51 | c.7542+13A>G | 0.026 |
| 51 | c.7543-156C>G | 0.078 |
| 52 | c.7661-61T>A | 0.013 |
| 54 | c.8027+11C>T | 0.143 |
| 57 | c.8547+153C>T | 0.208 |
| 58 | c.8668+209C>T | 0.026 |
| 58 | c.8669-75C>G | 0.039 |
| 61 | c.9163+189G>T | 0.052 |
| 64 | c.9361+138T>C | 0.169 |
| 64 | *c.9361+200C>T | 0.013 |
| 65 | c.9564-97C>T | 0.273 |
| 66 | c.9649+15 T>C | 0.325 |
| 67 | c.9807+218C>G | 0.026 |
| 68 | c.9974+13delA | 0.039 |
| 68 | c.9974+22dup | 0.091 |
| 68 | c.9974+22delA | 0.039 |
| 68 | c.9975-79G>A | 0.026 |
| 70 | c.10224-101T>C | 0.013 |
| 72 | c.10328+67A>G | 0.390 |
| 74 | c.10554-36_10554-33del | 0.325 |
| 75 | c.10797+82G>A | 0.052 |
| 75 | c.10798-100G>C | 0.039 |
